# Supportive care needs as an independent risk factor for survival in advanced liver cancer: a prospective cohort study

**DOI:** 10.3389/fonc.2026.1786118

**Published:** 2026-06-22

**Authors:** Xiaohui Liao, Dongping Liao, Hai Lin, Hongbin Chen

**Affiliations:** 1 Department of Gastroenterology, Sanming First Hospital Affiliated to Fujian Medical University, Sanming, China; 2 Department of Vascular Surgery, Sanming First Hospital Affiliated to Fujian Medical University, Sanming, China

**Keywords:** advanced cancer, influencing factors, liver cancer, prognosis, supportive care needs

## Abstract

**Background:**

Patients with advanced liver cancer often experience high unmet supportive care needs (SCN). However, longitudinal changes in SCN and their relationship with survival remain insufficiently understood.

**Objective:**

This study aimed to identify factors associated with longitudinal SCN and to examine the prognostic value of SCN in patients with advanced liver cancer.

**Methods:**

We enrolled 167 patients receiving transarterial chemoembolization (TACE) combined with targeted therapy and immunotherapy. The Hospital Anxiety and Depression Scale (HADS), Karnofsky Performance Status (KPS), Zarit Caregiver Burden Interview (ZBI), and Supportive Care Needs Survey (SCNS) were administered at baseline and at scheduled 6-month follow-up visits. Longitudinal SCNS total and domain scores were analyzed using random-slope linear mixed-effects models. Survival outcomes were evaluated using baseline Cox, time-dependent Cox, and lagged Cox models, with SCNS modeled as both a standardized continuous variable and categorical need groups.

**Results:**

Follow-up time, treatment response, HADS score, KPS score, ZBI score, education level, economic burden, and Child-Pugh grade were significantly associated with longitudinal SCNS total score. Progressive disease (PD) was associated with higher SCNS total score, whereas partial response (PR) was associated with lower SCNS total score. Domain-specific models showed that different SCN domains had distinct but overlapping determinants. In survival analyses, higher SCN was significantly associated with increased mortality. Each 1-SD increase in baseline SCNS total score was associated with higher mortality risk (HR = 1.95, 95% CI: 1.44–2.65). This association remained significant in the response-stratified time-dependent Cox model (HR = 3.17, 95% CI: 2.04–4.92) and in the lagged Cox model using previous-visit SCNS score to predict subsequent mortality (HR = 2.37, 95% CI: 1.64–3.42).

**Conclusion:**

SCN in advanced liver cancer is dynamic and influenced by multidimensional psychosocial and clinical factors. Higher SCNS total score was independently associated with poorer survival across baseline, time-dependent, and lagged survival models, suggesting that SCN may serve as a dynamic prognostic indicator. These findings support integrating repeated SCNS screening into routine clinical follow-up to facilitate early, individualized supportive care interventions.

## 1 Introduction

Liver cancer ranks among the most common malignancies worldwide and remains one of the leading causes of cancer-related death, posing a substantial global health burden (1, 2). Because of its aggressive disease course, complex treatment trajectory, and poor prognosis, patients with advanced liver cancer often experience multidimensional supportive care needs, including psychological, informational, physical, and care-related needs (3, 4). These clinical features may generate substantial supportive care needs (SCN), including psychological distress, symptom-related concerns, information needs, practical difficulties, and care coordination challenges.

SCN describe the perceived assistance required by individuals affected by cancer across diagnosis, treatment, follow-up, survivorship, palliative care, and bereavement (5–8). Rather than representing isolated patient preferences, unmet SCN may reflect a broader state of vulnerability shaped by symptom burden, psychological distress, functional decline, caregiver strain, treatment uncertainty, and barriers to accessing timely care. These factors are clinically important because they may influence treatment adherence, communication with healthcare professionals, use of supportive services, quality of life, and potentially survival outcomes (7–9). Therefore, longitudinal assessment of SCN may provide information that complements conventional tumor- and liver-function-based prognostic indicators.

Evidence specific to liver cancer remains limited. Previous studies have shown that patients with hepatocellular carcinoma may experience changing unmet needs during the transition from hospital to home, while qualitative research among patients undergoing interventional therapy has identified anxiety, uncertainty about treatment effects, insufficient understanding of procedures and adverse reactions, financial concerns, and needs for improved care processes (10, 11). A scoping review by the AASLD Practice Metrics Committee further supports the relevance of patient-reported outcomes in HCC care, particularly in relation to symptoms, treatment experience, quality of life, and survival (12). However, most existing evidence is cross-sectional, qualitative, or focused on patient-reported outcomes more broadly, and few studies have examined whether longitudinal SCN are associated with survival in patients with advanced liver cancer. This study therefore aimed to evaluate longitudinal SCN, identify associated psychosocial and clinical factors, and examine the relationship between SCN and survival outcomes in patients with advanced liver cancer.

## 2 Methods

### 2.1 Data collection

This was a single-center prospective cohort study conducted at Sanming First Hospital Affiliated to Fujian Medical University. Patients with advanced primary liver cancer who received TACE plus targeted therapy and immunotherapy between January 2022 and December 2023 were screened for eligibility. The specific inclusion and exclusion criteria were as follows:

#### 2.1.1 Inclusion criteria

1. Age ≥ 40 years, because the study focused on the predominant age range of advanced primary liver cancer in our center and aimed to reduce heterogeneity related to early-onset disease;
2. Diagnosed with primary liver cancer, with China Liver Cancer staging (CNLC) Stage IIIB, with portal vein tumor thrombus (PVTT) Type I or II, Child-Pugh Class A or B liver function, and a time since diagnosis of less than 3 months at the start of follow-up;
3. Treatment plan determined as TACE combined with targeted and immunotherapy after multidisciplinary team evaluation, with regular treatment received at our hospital. Regular treatment was defined as completion of at least 2 cycles of TACE therapy, continuous receipt of targeted and immunotherapy for at least 3 months, and no interruption of targeted therapy or immunotherapy exceeding 4 weeks, excluding planned intervals between TACE procedures. The 4-week threshold was used to ensure comparable longitudinal exposure to systemic treatment and response assessment.

#### 2.1.2 Exclusion criteria

1. Age < 40 years;
2. Diagnosed with secondary liver cancer;
3. Received combined or alternative treatments (surgical treatment, palliative care);
4. Time since diagnosis greater than 3 months at the start of follow-up;
5. Expected survival time less than 30 days, defined as severe clinical instability or end-stage condition judged by the treating physicians before enrollment, because questionnaire follow-up and combined therapy would not be clinically feasible in such patients;
6. Presence of other severe diseases that may affect survival prognosis: severe cardiac dysfunction (NYHA Class III–IV); severe renal dysfunction (eGFR < 60 mL/min/1.73m² or requiring renal replacement therapy); severe chronic obstructive pulmonary disease (GOLD Grade 3–4) or other severe pulmonary function disorders; severe neurological diseases (e.g., uncontrolled epilepsy, dementia, severe post-stroke disability); active autoimmune diseases requiring long-term immunosuppressive therapy; other uncontrolled malignancies;
7. Inability to obtain complete medical records and 2-year follow-up data.

### 2.2 Data organization

Based on the above criteria, a total of 218 cases of advanced liver cancer admitted to our hospital and receiving TACE combined with targeted and immunotherapy were included in this study. At the start of follow-up, the most recent AFP value was recorded, their Child-Pugh stage was assessed, and the tumor diameter at diagnosis was recorded. At enrollment, eligible patients completed a baseline questionnaire, including sociodemographic information, HADS, KPS, ZBI, and SCNS assessments. The SCNS is a widely used instrument for assessing cancer patients’ perceived SCN; the 34-item short form was developed and validated to reduce respondent burden while retaining multidimensional assessment of SCN (13). The questionnaire information from patients was recorded and quantified (The specific scales and quantification methods are shown in **Supplementary Tables 1**–**4**).

Patients were followed for up to 24 months after enrollment. Questionnaire-based assessments, including the HADS, KPS, ZBI, and SCNS, were scheduled at baseline and every 6 months thereafter. Survival status and survival time were recorded for all included patients. Patients alive at the end of follow-up were censored at the last follow-up date, whereas patients who died during follow-up were recorded as events, with survival time calculated in months from enrollment to death.

Tumor response was obtained from hospital medical records and radiological efficacy evaluations during follow-up. Radiological response was assessed according to RECIST version 1.1 and classified as complete response (CR), partial response (PR), stable disease (SD), or progressive disease (PD). For each scheduled questionnaire visit, the corresponding tumor response status was abstracted from the medical record and entered into the same patient-visit record as SCNS, HADS, KPS, and ZBI.

### 2.3 Statistical analysis

Descriptive statistics were used to summarize baseline characteristics and longitudinal follow-up data. Categorical variables are presented as numbers and percentages, and continuous questionnaire scores are presented as means with standard deviations. Between-group comparisons were performed using the chi-square test or Fisher’s exact test, as appropriate.

Because SCNS, HADS, KPS, and ZBI were repeatedly assessed within the same patients, longitudinal SCN were analyzed using linear mixed-effects models. The primary longitudinal outcome was the SCNS total score. The primary model was specified *a priori* to include follow-up time, treatment response, HADS score, KPS score, ZBI score, education level, economic burden, AFP level, tumor diameter, and Child-Pugh grade as fixed effects, with patient-specific random intercepts and random slopes for follow-up time. These variables were selected because they were considered to have direct clinical or theoretical relevance to longitudinal SCN, reflecting psychological distress, functional status, caregiver burden, treatment response, socioeconomic context, and tumor-related disease burden.

To avoid overfitting and unstable estimation in a relatively small cohort, the primary mixed-effects model was not expanded to include all available demographic and social variables simultaneously. Although repeated measurements increased the number of visit-level observations, the effective number of independent participants remained 167, and the random-slope model already required estimation of both fixed effects and patient-level random effects. Therefore, a parsimonious primary model was used to preserve model stability and interpretability.

To evaluate the robustness of the findings, an extended fully adjusted sensitivity model was additionally fitted. This extended model further included age, sex, BMI, marital status, religious belief, and place of residence, in addition to all covariates in the primary model. The same modeling strategy was applied to the five SCNS domain scores. Generalized estimating equations with an exchangeable working correlation structure were used as an additional sensitivity analysis for the SCNS total score. Survival analyses were performed using Cox proportional hazards models. First, a baseline Cox model was fitted using baseline SCNS total score. Second, an extended time-dependent Cox model was constructed using start–stop intervals, in which SCNS total score, HADS, KPS, and ZBI were updated at each questionnaire follow-up. The SCNS total score was standardized, and hazard ratios were reported per 1-SD increase. Treatment response was incorporated as a stratification variable in the time-dependent Cox model to allow different baseline hazards across response categories and to avoid unstable estimates caused by sparse events in some response groups. Robust standard errors clustered by patient ID were used to account for repeated risk intervals within the same patient.

To reduce potential reverse causation, a lagged Cox model was further fitted, in which the SCNS score from the previous visit was used to predict subsequent mortality. As additional sensitivity analyses, SCNS was analyzed as a four-level categorical variable, with the low-need group used as the reference category. The proportional hazards assumption was assessed using Schoenfeld residuals. Variables showing evidence of non-proportional hazards were interpreted cautiously. All statistical tests were two-sided, and p<0.05 was considered statistically significant. Analyses were performed using R software(version 4.4.2).

## 3 Results

### 3.1 Descriptive analysis of the included sample

A total of 167 patients with advanced liver cancer receiving TACE combined with targeted and immunotherapy were included, providing 667 valid visit-level observations for longitudinal modeling. The numbers of available observations at baseline, 6 months, 12 months, 18 months, and 24 months were 167, 163, 148, 114, and 75, respectively. The mean SCNS total score was 59.1 ± 14.6 at baseline, 59.6 ± 15.6 at 6 months, 60.7 ± 16.0 at 12 months, 59.1 ± 15.3 at 18 months, and 55.1 ± 14.8 at 24 months. HADS and ZBI scores showed a gradual increase during follow-up, whereas the number of patients remaining under observation decreased over time because of death or censoring.

At baseline, patients were categorized into low, medium-low, medium-high, and high supportive care need groups according to SCNS total score. Baseline characteristics differed across SCNS need groups, particularly in terms of age, tumor diameter, AFP level, HADS score, KPS score, and ZBI score. The high-need group had a higher proportion of patients aged ≤65 years than the low-need group (73.81% vs 41.86%). Patients in the high-need group also had higher proportions of tumor diameter >10 cm (64.29%) and AFP >400 ng/mL (71.43%). High caregiver burden was more frequent in the high-need group than in the low-need group (57.14% vs 23.26%). Because **Table 1** provides omnibus comparisons across four SCNS groups, pairwise group differences were not inferred solely from p values (**Tables 1**, **2**).

**Table 1:** Basic clinical information of groups with different degrees of SCN.

Variable	Total (n=167)	Low (n=43)	Medium-low (n=41)	Medium-high (n=41)	High (n=42)	χ² statistic	P value
Gender, n(%)						χ²=4.23	0.237
Female	69 (41.32)	21 (48.84)	16 (39.02)	12 (29.27)	20 (47.62)		
Male	98 (58.68)	22 (51.16)	25 (60.98)	29 (70.73)	22 (52.38)		
Age, n(%)						χ²=14.36	0.002
≤65	91 (54.49)	18 (41.86)	16 (39.02)	26 (63.41)	31 (73.81)		
>65	76 (45.51)	25 (58.14)	25 (60.98)	15 (36.59)	11 (26.19)		
BMI, n(%)						χ²=2.53	0.469
Normal range	75 (44.91)	23 (53.49)	15 (36.59)	19 (46.34)	18 (42.86)		
Below the normal range	92 (55.09)	20 (46.51)	26 (63.41)	22 (53.66)	24 (57.14)		
Marital status, n(%)						χ²=2.43	0.488
Unmarried/divorced	32 (19.16)	11 (25.58)	8 (19.51)	5 (12.20)	8 (19.05)		
Married	135 (80.84)	32 (74.42)	33 (80.49)	36 (87.80)	34 (80.95)		
Education, n(%)						χ²=19.76	0.072
Illiteracy	50 (29.94)	13 (30.23)	14 (34.15)	14 (34.15)	9 (21.43)		
Primary school	39 (23.35)	11 (25.58)	11 (26.83)	8 (19.51)	9 (21.43)		
Junior high school	30 (17.96)	12 (27.91)	6 (14.63)	9 (21.95)	3 (7.14)		
Senior high school	25 (14.97)	4 (9.30)	4 (9.76)	4 (9.76)	13 (30.95)		
Undergraduate college	23 (13.77)	3 (6.98)	6 (14.63)	6 (14.63)	8 (19.05)		
Religion, n(%)						χ²=7.14	0.068
No religion	87 (52.10)	19 (44.19)	27 (65.85)	24 (58.54)	17 (40.48)		
Possess religious beliefs	80 (47.90)	24 (55.81)	14 (34.15)	17 (41.46)	25 (59.52)		
Place of residence, n(%)						χ²=5.83	0.120
Rural areas	78 (46.71)	17 (39.53)	23 (56.10)	23 (56.10)	15 (35.71)		
Cities and towns	89 (53.29)	26 (60.47)	18 (43.90)	18 (43.90)	27 (64.29)		
Economic burden, n(%)						χ²=9.29	0.158
Low	58 (34.73)	7 (16.28)	17 (41.46)	16 (39.02)	18 (42.86)		
Medium	45 (26.95)	15 (34.88)	11 (26.83)	9 (21.95)	10 (23.81)		
High	64 (38.32)	21 (48.84)	13 (31.71)	16 (39.02)	14 (33.33)		
Tumor diameter, n(%)						χ²=15.98	0.001
≤10cm	92 (55.09)	21 (48.84)	32 (78.05)	24 (58.54)	15 (35.71)		
>10cm	75 (44.91)	22 (51.16)	9 (21.95)	17 (41.46)	27 (64.29)		
AFP, n(%)						χ²=13.57	0.004
≤400ng/mL	68 (40.72)	18 (41.86)	26 (63.41)	12 (29.27)	12 (28.57)		
>400ng/mL	99 (59.28)	25 (58.14)	15 (36.59)	29 (70.73)	30 (71.43)		
Child-Pugh, n(%)						χ²=7.09	0.069
Grade A	95 (56.89)	21 (48.84)	30 (73.17)	24 (58.54)	20 (47.62)		
Grade B	72 (43.11)	22 (51.16)	11 (26.83)	17 (41.46)	22 (52.38)		
Hads, n(%)						χ²=74.34	<.001
Low	40 (23.95)	12 (27.91)	14 (34.15)	7 (17.07)	7 (16.67)		
Medium-low	40 (23.95)	4 (9.30)	15 (36.59)	11 (26.83)	10 (23.81)		
Medium-severe	42 (25.15)	0 (0.00)	3 (7.32)	16 (39.02)	23 (54.76)		
Severe	45 (26.95)	27 (62.79)	9 (21.95)	7 (17.07)	2 (4.76)		
KPS, n(%)						χ²=15.89	0.014
90–100 points	65 (38.92)	13 (30.23)	15 (36.59)	17 (41.46)	20 (47.62)		
70–80 points	47 (28.14)	7 (16.28)	13 (31.71)	17 (41.46)	10 (23.81)		
50–60 points	55 (32.93)	23 (53.49)	13 (31.71)	7 (17.07)	12 (28.57)		
Zarit, n(%)						χ²=19.01	0.004
Low	57 (34.13)	18 (41.86)	21 (51.22)	11 (26.83)	7 (16.67)		
Medium	45 (26.95)	15 (34.88)	9 (21.95)	10 (24.39)	11 (26.19)		
High	65 (38.92)	10 (23.26)	11 (26.83)	20 (48.78)	24 (57.14)		
Physiological and daily life needs, n(%)						χ²=6.01	0.422
Low	44 (26.35)	14 (32.56)	11 (26.83)	12 (29.27)	7 (16.67)		
Medium	51 (30.54)	16 (37.21)	12 (29.27)	10 (24.39)	13 (30.95)		
High	72 (43.11)	13 (30.23)	18 (43.90)	19 (46.34)	22 (52.38)		
Psychological needs,n(%)						χ²=48.76	<.001
Low	54 (32.34)	28 (65.12)	15 (36.59)	9 (21.95)	2 (4.76)		
Medium	60 (35.93)	9 (20.93)	18 (43.90)	19 (46.34)	14 (33.33)		
High	53 (31.74)	6 (13.95)	8 (19.51)	13 (31.71)	26 (61.90)		
Sexual needs, n(%)						χ²=10.20	0.116
Low	85 (50.90)	28 (65.12)	24 (58.54)	17 (41.46)	16 (38.10)		
Medium	50 (29.94)	11 (25.58)	11 (26.83)	14 (34.15)	14 (33.33)		
High	32 (19.16)	4 (9.30)	6 (14.63)	10 (24.39)	12 (28.57)		
Patient care and support needs, n(%)						χ²=24.02	<.001
Low	46 (27.54)	15 (34.88)	19 (46.34)	10 (24.39)	2 (4.76)		
Medium	30 (17.96)	8 (18.60)	9 (21.95)	6 (14.63)	7 (16.67)		
High	91 (54.49)	20 (46.51)	13 (31.71)	25 (60.98)	33 (78.57)		
Health systems and information needs,n(%)						–	<.001*
Low	45 (26.95)	29 (67.44)	10 (24.39)	6 (14.63)	0 (0.00)		
Medium	15 (8.98)	6 (13.95)	4 (9.76)	2 (4.88)	3 (7.14)		
High	107 (64.07)	8 (18.60)	27 (65.85)	33 (80.49)	39 (92.86)		

χ², Chi-square test; -, Fisher exact; *, Simulated p-value.

Data are presented as n (%). P values were calculated using chi-square tests or Fisher’s exact tests, as appropriate, and represent omnibus comparisons across the four SCNS need groups. No *post-hoc* pairwise comparisons were performed; therefore, Bonferroni correction was not applied. Economic burden was assessed using the baseline sociodemographic questionnaire and categorized according to patients’ self-reported perceived financial burden as low, medium, or high. SCNS=Supportive Care Needs Survey; AFP=alpha-fetoprotein; HADS=Hospital Anxiety and Depression Scale; KPS=Karnofsky Performance Status.

**Table 2:** Longitudinal distribution of SCN and related patient-reported measures during 24-month follow-up.

Follow-up time, months	No. of records	SCNS total score, mean (SD)	HADS, mean (SD)	KPS, mean (SD)	Zarit, mean (SD)
0	167	59.1 (14.6)	1.55 (1.13)	0.94 (0.85)	1.05 (0.86)
6	163	59.6 (15.6)	1.80 (1.16)	1.01 (0.80)	1.17 (0.84)
12	148	60.7 (16.0)	1.99 (1.13)	0.87 (0.80)	1.36 (0.82)
18	114	59.1 (15.3)	2.06 (1.11)	0.75 (0.78)	1.46 (0.78)
24	75	55.1 (14.8)	1.99 (1.16)	0.76 (0.75)	1.41 (0.76)

Data are presented as mean (SD) unless otherwise indicated. The decreasing number of records over time reflects death or censoring during follow-up. SCN, supportive care needs; SCNS, Supportive Care Needs Survey; HADS, Hospital Anxiety and Depression Scale; KPS, Karnofsky Performance Status; Zarit, Zarit Caregiver Burden Interview; SD, standard deviation.

### 3.2 Factors influencing SCN in advanced liver cancer patients

In the random-slope linear mixed-effects model for longitudinal SCNS total score (**Table 3**), the random-slope model fitted the data significantly better than the random-intercept-only model, and no singular fit was detected. Follow-up time was positively associated with SCNS total score, with an average increase of 1.09 points for every 6-month increase in follow-up time (β=1.09, 95% CI 0.76 to 1.41, p<0.001).

**Table 3:** Longitudinal determinants of SCNS total score in patients with advanced liver cancer: random-slope linear mixed-effects model.

Variable	β (95% CI)	P value
Follow-up time, per 6 months	1.09 (0.76 to 1.41)	<0.001
Treatment response: PR	-1.46 (-2.08 to -0.85)	<0.001
Treatment response: SD	-0.42 (-0.86 to 0.02)	0.061
Treatment response: PD	2.53 (1.82 to 3.24)	<0.001
HADS score	1.20 (0.71 to 1.69)	<0.001
KPS score	-2.52 (-3.02 to -2.01)	<0.001
Zarit caregiver burden score	0.90 (0.45 to 1.35)	<0.001
Education level		
Primary school	0.51 (-4.95 to 5.96)	0.854
Junior high school	-2.57 (-8.67 to 3.52)	0.406
Senior high school	11.71 (5.30 to 18.12)	<0.001
Undergraduate college	6.56 (0.03 to 13.10)	0.049
Economic burden		
Medium	-8.46 (-13.55 to -3.38)	0.001
High	-8.29 (-13.32 to -3.25)	0.001
AFP(>400ng/mL)	5.46 (-0.12 to 11.05)	0.055
Tumor diameter(>10cm)	6.66 (-0.45 to 13.78)	0.066
Child-Pugh(Grade B)	-8.19 (-15.27 to -1.10)	0.024

β coefficients represent the estimated change in SCNS total score associated with each variable in the random-slope linear mixed-effects model. Follow-up time was modeled per 6-month increase. Treatment response was included as a time-updated covariate, with non-evaluable baseline records used as the reference category. For categorical variables, the reference categories were illiteracy for education level, low economic burden for economic burden, AFP ≤400 ng/mL, tumor diameter ≤10 cm, and Child-Pugh grade A. PR, partial response; SD, stable disease; PD, progressive disease; SCNS, Supportive Care Needs Survey; HADS, Hospital Anxiety and Depression Scale; KPS, Karnofsky Performance Status; AFP, alpha-fetoprotein; CI, confidence interval.

According to RECIST version 1.1, treatment response was classified as complete response (CR), partial response (PR), stable disease (SD), or progressive disease (PD). No CR records were observed during follow-up; therefore, PR, SD, and PD were included as response categories in the longitudinal model. Compared with non-evaluable baseline records, PR was associated with lower SCNS total score (β=-1.46, 95% CI -2.08 to -0.85, p<0.001), whereas PD was associated with higher SCNS total score (β=2.53, 95% CI 1.82 to 3.24, p<0.001). HADS score (β=1.20, 95% CI 0.71 to 1.69, p<0.001) and ZBI score (β=0.90, 95% CI 0.45 to 1.35, p<0.001) were positively associated with SCNS total score, whereas KPS score was negatively associated with SCNS total score (β=-2.52, 95% CI -3.02 to -2.01, p<0.001). Education level and economic burden were also associated with longitudinal SCNS total score. The GEE sensitivity analysis showed broadly consistent associations for HADS, KPS, ZBI, treatment response, education level, and economic burden.

### 3.3 Factors influencing SCN subgroups in advanced liver cancer patients

Separate linear mixed-effects models were fitted for the five SCNS domains. For physical and daily living needs, PD, HADS score, and ZBI score were positively associated with higher needs, whereas KPS score was negatively associated with this domain. For psychological needs, follow-up time, PD, HADS score, education level, and AFP level were positively associated with higher needs, whereas PR and higher KPS score were associated with lower psychological needs.

**Figure 1:**
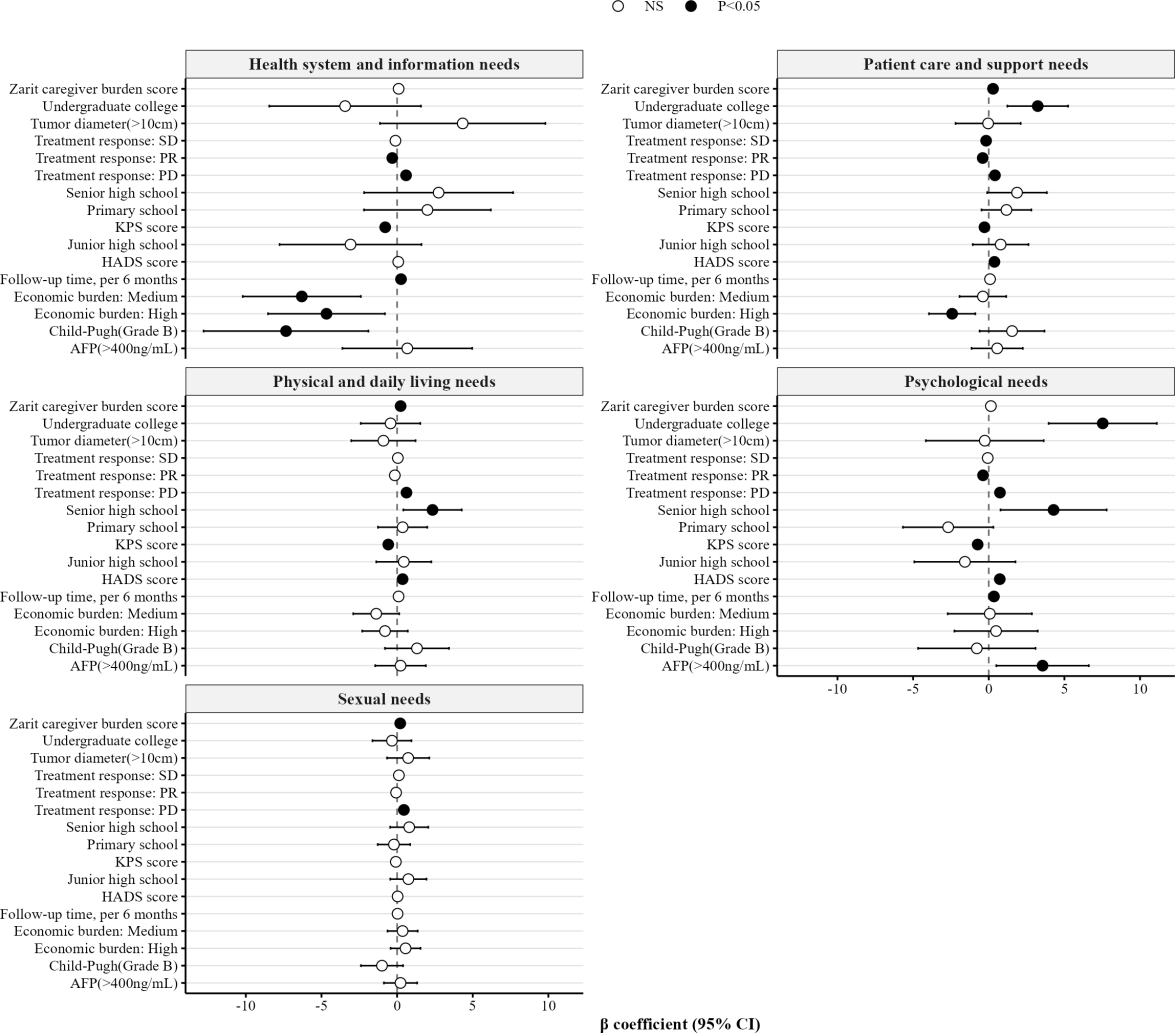
Domain-specific longitudinal determinants of SCN in patients with advanced liver cancer.

For sexual needs, PD and higher ZBI score were associated with increased needs, suggesting that sexual concerns were not entirely independent of disease progression and caregiver burden. For patient care and support needs, treatment response, HADS score, KPS score, ZBI score, education level, and economic burden were significant correlates. PR and SD were associated with lower care/support needs, whereas PD was associated with higher care/support needs. For health system and information needs, follow-up time, PD, KPS score, economic burden, and Child-Pugh grade were significant correlates. These findings suggest that different SCNS domains have distinct but overlapping clinical and psychosocial drivers (**Figure 1**).

Estimates are β coefficients from separate random-slope linear mixed-effects models for each SCNS domain. Positive coefficients indicate higher domain scores, whereas negative coefficients indicate lower domain scores. Tumor response was assessed according to RECIST version 1.1; CR was not observed during follow-up. CR=complete response; PR=partial response; SD=stable disease; PD=progressive disease; SCNS=Supportive Care Needs Survey; CI=confidence interval.

To further evaluate the robustness of the longitudinal findings, extended fully adjusted sensitivity models were fitted by additionally adjusting for age, sex, BMI, marital status, religious belief, and place of residence. The extended random-slope linear mixed-effects model for SCNS total score yielded results broadly consistent with the primary model. Follow-up time remained positively associated with SCNS total score (β=1.08, 95% CI 0.76 to 1.40, p<0.001). PR remained associated with lower SCNS total score (β=-1.47, 95% CI -2.08 to -0.86, p<0.001), whereas PD remained associated with higher SCNS total score (β=2.52, 95% CI 1.81 to 3.23, p<0.001). HADS score, KPS score, ZBI score, education level, and economic burden also showed associations consistent with the primary model. Age >65 years was additionally associated with lower SCNS total score in the extended model (β=-6.08, 95% CI -10.09 to -2.06, p=0.003). In the extended domain-specific models, the major associations observed in the primary analyses were generally preserved across physical and daily living, psychological, sexual, patient care/support, and health system/information needs. All extended models were successfully fitted as random-slope models without singular fit. Detailed results are provided in **Supplementary Tables 6**–**8**.

### 3.4 Impact of SCN on the prognosis of advanced liver cancer patients

In the baseline Cox model, higher baseline SCNS total score was significantly associated with increased mortality. Each 1-SD increase in baseline SCNS total score was associated with a 1.95-fold higher risk of death (HR = 1.95, 95% CI 1.44 to 2.65, p<0.001) (**Table 4**). When SCNS was analyzed as a categorical variable, the low-need group was used as the reference group. Compared with the low-need group, the medium-low-need group had a significantly higher mortality risk (HR = 4.77, 95% CI 2.01 to 11.33, p<0.001), as did the medium-high-need group (HR = 4.26, 95% CI 1.75 to 10.39, p=0.001) and the high-need group (HR = 7.71, 95% CI 3.20 to 18.56, p<0.001) (**Table 5**).

**Table 4:** Association of continuous SCNS total score with mortality across baseline, time-dependent, and lagged Cox models.

Model	Comparison or unit	HR (95% CI)	P value
Baseline Cox	Per 1-SD increase	1.95 (1.44 to 2.65)	<0.001
Time-dependent Cox	Per 1-SD increase	3.17 (2.04 to 4.92)	<0.001
Lagged Cox	Per 1-SD increase	2.37 (1.64 to 3.42)	<0.001

Hazard ratios are reported per 1-SD increase in SCNS total score. In the time-dependent Cox model, SCNS and other patient-reported measures were updated at each scheduled follow-up visit. In the lagged Cox model, the previous-visit SCNS value was used to predict subsequent mortality. HR, hazard ratio; CI, confidence interval; SCNS, Supportive Care Needs Survey.

**Table 5:** Association of baseline SCNS need group with mortality.

Comparison	HR (95% CI)	P value
Medium-low vs Low	4.77 (2.01 to 11.33)	<0.001
Medium-high vs Low	4.26 (1.75 to 10.39)	0.001
High vs Low	7.71 (3.20 to 18.56)	<0.001

Estimates were derived from the baseline Cox model. The low-need group was used as the reference category. HR, hazard ratio; CI, confidence interval; SCNS, Supportive Care Needs Survey.

In the response-stratified time-dependent Cox model, 580 risk intervals and 92 death events were included. After adjustment for HADS, KPS, ZBI, education level, economic burden, AFP level, tumor diameter, and Child-Pugh grade, each 1-SD increase in time-updated SCNS total score was associated with a 3.17-fold higher risk of death (HR = 3.17, 95% CI 2.04 to 4.92, p<0.001) (**Table 4**). Higher time-updated KPS score was associated with lower mortality risk (HR = 0.49, 95% CI 0.35 to 0.69, p<0.001), whereas higher ZBI score was associated with increased mortality risk (HR = 1.90, 95% CI 1.29 to 2.80, p=0.001).

The categorical time-dependent Cox model showed a similar pattern. Compared with the low-need group, the medium-high and high-need groups had significantly increased mortality risks, with HRs of 8.14 (95% CI 1.68 to 39.50, p=0.009) and 13.97 (95% CI 2.70 to 72.40, p=0.002), respectively. The medium-low group showed a higher but non-significant risk estimate (**Table 6**).

**Table 6:** Association of time-updated SCNS need group with mortality in the response-stratified time-dependent Cox model.

Comparison	HR (95% CI)	P value
Medium-low vs Low	2.69 (0.54 to 13.43)	0.227
Medium-high vs Low	8.14 (1.68 to 39.50)	0.009
High vs Low	13.97 (2.70 to 72.40)	0.002

The low-need group was used as the reference category. SCNS need group and other patient-reported measures were updated at each scheduled follow-up visit. Treatment response was incorporated as a stratification variable in the time-dependent Cox model. HR, hazard ratio; CI, confidence interval; SCNS, Supportive Care Needs Survey.

In the lagged Cox model, 413 risk intervals and 87 death events were analyzed. The previous-visit SCNS total score remained significantly associated with subsequent mortality. Each 1-SD increase in previous SCNS total score was associated with a 2.37-fold higher subsequent mortality risk (HR = 2.37, 95% CI 1.64 to 3.42, p<0.001) (**Table 4**). When previous SCNS need group was analyzed categorically, the medium-low, medium-high, and high-need groups all had significantly higher subsequent mortality risks than the low-need group. These lagged analyses support the robustness of the association between SCN and mortality and reduce the likelihood that the observed association was solely driven by terminal deterioration (**Table 7**).

**Table 7:** Association of previous-visit SCNS need group with subsequent mortality in the lagged Cox model.

Comparison	HR (95% CI)	P value
Medium-low vs Low	6.45 (1.85 to 22.44)	0.003
Medium-high vs Low	10.39 (2.89 to 37.30)	<0.001
High vs Low	17.36 (4.74 to 63.60)	<0.001

The low-need group was used as the reference category. Previous-visit SCNS need group was used to predict mortality during the subsequent interval. HR, hazard ratio; CI, confidence interval; SCNS, Supportive Care Needs Survey.

Proportional hazards testing showed that the SCNS exposure did not significantly violate the proportional hazards assumption in either the time-dependent Cox model or the lagged Cox model. Some covariates showed evidence of non-proportional hazards, and their HRs were therefore interpreted cautiously (**Supplementary Table 5**).

## 4 Discussion

### 4.1 Association between SCN and prognosis in advanced liver cancer patients

The principal finding of this study is that SCN were not only dynamic during follow-up but also prognostically meaningful in patients with advanced liver cancer. The consistency of the baseline, response-stratified time-dependent, and lagged Cox models suggests that repeated SCNS assessments may capture a clinically relevant vulnerability that is not limited to baseline symptom burden or terminal deterioration.

Across cancer populations, unmet SCN commonly involve psychological, physical, informational, practical, and caregiver-related domains, with patterns varying by diagnosis and care context (7, 9, 14–19). Prior work has also linked unmet SCN with clinically meaningful outcomes such as emergency department use and hospitalization, suggesting that needs assessments may capture patient vulnerability beyond quality-of-life impairment alone (19).

Cancer prognosis is influenced by a multitude of factors, among which psychosocial elements and patient-reported outcomes may provide prognostic information beyond conventional clinical indicators. Previous studies have suggested that patient-reported health-related quality-of-life measures can predict survival in cancer populations, including hepatobiliary cancer (20, 21). In HCC specifically, patient-reported outcome research has increasingly emphasized the relationship between symptoms, quality of life, treatment experience, supportive and palliative care needs, and survival (12, 22, 23). The HCC-focused supportive care literature also highlights early attention to symptom burden, psychosocial distress, communication, and caregiver support alongside disease-directed therapy (23).

### 4.2 Multidimensional factors influencing SCN

SCN are influenced by multiple factors, and early identification of relevant factors is crucial for predicting patient needs and implementing timely interventions. In the revised longitudinal analysis, random-slope linear mixed-effects models confirmed that follow-up time, treatment response, psychological status (HADS score), functional status (KPS score), caregiver burden (Zarit score), education level, economic burden, and selected tumor-related factors were associated with SCN over time. Compared with the original cross-sectional or averaging-based approach, this longitudinal model better accounts for within-patient correlation and individual changes during follow-up.

Patients with higher education levels exhibited more prominent needs in the psychological and medical information dimensions, potentially stemming from their deeper disease awareness and stronger information-seeking capabilities (24, 25). A positive correlation was observed between anxiety/depression status (HADS score) and SCN, particularly psychological and care support needs, highlighting the significant value of psychological intervention (26). This is consistent with NCCN distress management guidance, which emphasizes the identification and treatment of psychosocial problems in patients with cancer (27). Poorer functional status, reflected by lower KPS score, was associated with higher physical, psychological, care/support, and health system/information needs. Heavier caregiver burden (higher Zarit score) positively correlated with multiple patient needs; studies indicate a significant association with psychological support and symptom management needs (28). The complex symptoms of liver cancer and frequent medical interventions exacerbate the physical and psychological burden on caregivers (29, 30), while enhancing family resilience can buffer the negative impact of this burden on needs (31), suggesting that caregivers should be integrated into holistic support plans.

The influencing factors varied across different need domains. Psychological needs were associated with follow-up time, treatment response, HADS score, KPS score, education level, and AFP level, underscoring the necessity for repeated psychological screening, particularly in patients with psychological distress, functional decline, or disease progression. Health system and information needs were associated with follow-up time, treatment response, KPS score, economic burden, and Child-Pugh grade, suggesting that informational needs may evolve dynamically during disease management rather than being determined only by baseline tumor burden. For physical and daily living needs, disease progression, psychological distress, functional status, and caregiver burden were relevant factors, indicating that symptom burden and family care capacity should be considered together. Sexual needs were associated with disease progression and caregiver burden, suggesting that this domain should not be overlooked even in advanced disease. Patient care and support needs were influenced by treatment response, psychological status, functional status, caregiver burden, education level, and economic burden, reflecting the critical role of both clinical and socioeconomic factors. Overall, these domain-specific findings support stratified and dynamically updated supportive care strategies. Repeated SCNS screening may help identify patients whose needs increase over time or after disease progression, thereby enabling timely psychological support, symptom management, caregiver assistance, information provision, and financial counseling. This approach is consistent with recent international efforts to integrate systematic, needs-based supportive care into routine oncology services (32). It is also aligned with ASCO recommendations that palliative care should be integrated into standard oncology care for patients with cancer, particularly those with advanced disease or substantial symptom burden (33). Therefore, repeated needs assessment may help allocate supportive resources more precisely according to evolving patient need profiles.

### 4.3 Limitation

As a single-center prospective study with a relatively limited sample size—and given that all enrolled patients received TACE combined with targeted and immunotherapy—the generalizability of the conclusions requires caution. Although longitudinal mixed-effects models and time-dependent Cox models were used to account for repeated measurements and time-updated exposures, the influence of unmeasured factors, such as social support, treatment adherence, nutritional status, symptom burden, treatment-related adverse events, and access to community resources, cannot be fully excluded.

Death during follow-up led to a decreasing number of available questionnaire observations over time. Although post-death records were excluded and mixed-effects models can accommodate unbalanced repeated measurements, informative dropout caused by disease progression or death may still influence longitudinal estimates. In addition, tumor response and questionnaire assessments were aligned according to scheduled 6-month follow-up intervals. Because tumor response assessments in clinical practice may occur at shorter or irregular intervals, future studies with exact assessment dates may allow more refined time-varying modeling.

The proportional hazards assumption was not fully satisfied for some covariates in the survival models. However, the main SCNS exposure did not show significant violation of the proportional hazards assumption in the time-dependent and lagged Cox models, and therefore the SCNS-related HRs were considered interpretable. HRs for covariates with potential non-proportional hazards should be interpreted cautiously.

Finally, this observational study demonstrates association rather than causality. Although the lagged Cox model reduced the likelihood of reverse causation, interventional studies are still needed to determine whether actively identifying and reducing SCN can improve survival, quality of life, treatment adherence, or other clinically meaningful outcomes in patients with advanced liver cancer.

## 5 Conclusion

Longitudinal SCNS assessments were associated with progressively increasing mortality risk and had several identified clinical and psychosocial cofactors. Further research should focus on exploring repeated SCNS assessments and assessing whether targeted psychosocial interventions can improve mortality and other clinically meaningful outcomes in this population.

## Data Availability

The raw data supporting the conclusions of this article will be made available by the authors, without undue reservation.
