# Supplementary material for: Supportive care needs as an independent risk factor for survival in advanced liver cancer: a prospective cohort study
Source: Front Oncol. 2026 Jun 22;16:1786118. doi: 10.3389/fonc.2026.1786118 (PMC13333445; doi:10.3389/fonc.2026.1786118)
Supplement: Supplementary file 1 [file Table1.docx]

****Supplementary Table 1:Karnofsky Performance Status (KPS) score****

| **clinical symptoms** | ****score**** |
| --- | --- |
| Normal; no complaints; no evidence of disease. | 100 |
| Able to carry on normal activity; minor signs or symptoms of disease. | 90 |
| Cares for self; unable to carry on normal activity or to do active work. | 80 |
| Cares for most personal needs; requires occasional assistance. | 70 |
| Requires considerable assistance and frequent medical care. | 60 |
| Disabled; requires special care and assistance. | 50 |
| Severely disabled; hospitalization is indicated, although death not imminent. | 40 |
| Very sick; hospitalization necessary; requires active supportive treatment. | 30 |
| Moribund; fatal processes progressing rapidly. | 20 |
| Critical condition, close to death. | 10 |
| Dead. | 0 |

### The Karnofsky Performance Status (KPS) scale is a standardized, clinician-rated instrument widely used to quantify a patient's functional status and ability to carry out daily activities. It employs an 11-point scale ranging from 100 (normal, no complaints, no evidence of disease) to 0 (death), with decrements of 10. Higher scores indicate a greater capacity for self-care and normal activity. The KPS is a valuable tool for prognostic stratification, treatment decision-making, and monitoring clinical change over time in patients, particularly those with chronic or debilitating illnesses like cancer^[1]^.

### ****Supplementary Table 2:Hospital Anxiety and Depression Scale (HADS)****^[2]^

****Instructions for patients:****

This questionnaire is designed to help your doctor know how you have been feeling over the past week. Please read each item and place a check mark (√) in the box opposite the reply that comes closest to how you have been feeling. Don't take too long over your replies; your immediate reaction to each item will probably be more accurate than a long, thought-out response.

| Item No. | Question | Choose the reply that best describes how you have been feeling in the past week |
| --- | --- | --- |
|  |  | ****Response Options**** |
| ****1**** | ****I feel tense or ‘wound up’:**** | □ Most of the time □ A lot of the time □ From time to time, occasionally □ Not at all |
| ****2**** | ****I still enjoy the things I used to enjoy:**** | □ Definitely as much □ Not quite so much □ Only a little □ Hardly at all |
| ****3**** | ****I get a sort of frightened feeling as if something awful is about to happen:**** | □ Very definitely and quite badly □ Yes, but not too badly □ A little, but it doesn't worry me □ Not at all |
| ****4**** | ****I can laugh and see the funny side of things:**** | □ As much as I always could □ Not quite so much now □ Definitely not so much now □ Not at all |
| ****5**** | ****Worrying thoughts go through my mind:**** | □ A great deal of the time □ A lot of the time □ From time to time, but not too often □ Only occasionally |
| ****6**** | ****I feel cheerful:**** | □ Not at all □ Not often □ Sometimes □ Most of the time |
| ****7**** | ****I can sit at ease and feel relaxed:**** | □ Definitely □ Usually □ Not Often □ Not at all |
| ****8**** | ****I feel as if I am slowed down:**** | □ Nearly all the time □ Very often □ Sometimes □ Not at all |
| ****9**** | ****I get a sort of frightened feeling like 'butterflies' in the stomach:**** | □ Not at all □ Occasionally □ Quite Often □ Very Often |
| ****10**** | ****I have lost interest in my appearance:**** | □ Definitely □ I don't take as much care as I should □ I may not take quite as much care □ I take just as much care as ever |
| ****11**** | ****I feel restless as if I have to be on the move:**** | □ Very much indeed □ Quite a lot □ Not very much □ Not at all |
| ****12**** | ****I look forward with enjoyment to things:**** | □ As much as I ever did □ Rather less than I used to □ Definitely less than I used to □ Hardly at all |
| ****13**** | ****I get sudden feelings of panic:**** | □ Very often indeed □ Quite often □ Not very often □ Not at all |
| ****14**** | ****I can enjoy a good book or radio or TV program:**** | □ Often □ Sometimes □ Not often □ Very seldom |

### ****Scoring and Interpretation****

****Scoring Key:****

Each item is scored on a scale of 0 to 3. The scoring differs for anxiety (A) and depression (D) items, and some are reverse-scored.

****Anxiety Scale (A) - Items 1, 3, 5, 7, 9, 11, 13****

or items 1, 3, 5, 9, 11, 13: ****Most of the time / Very...**** = 3, ****A lot of the time / Quite...**** = 2, ****From time to time / Not very...**** = 1, ****Not at all / Occasionally**** = 0.

For item 7 (reverse-scored): ****Definitely**** = 0, ****Usually**** = 1, ****Not Often**** = 2, ****Not at all**** = 3.

****Depression Scale (D) - Items 2, 4, 6, 8, 10, 12, 14****

For items 2, 4, 12, 14 (reverse-scored): ****Definitely as much / As much as I ever did / Often**** = 0, ****Not quite so much / Sometimes**** = 1, ****Only a little / Definitely less / Not often**** = 2, ****Hardly at all / Very seldom**** = 3.

For items 6 (reverse-scored): ****Most of the time**** = 0, ****Sometimes**** = 1, ****Not often**** = 2, ****Not at all**** = 3.

For item 8: ****Nearly all the time**** = 3, ****Very often**** = 2, ****Sometimes**** = 1, ****Not at all**** = 0.

For item 10: ****Definitely**** = 3, ****I don't take as much care as I should**** = 2, ****I may not take quite as much care**** = 1, ****I take just as much care as ever**** = 0.

****Interpretation of Total Scores:****

For both the Anxiety (HADS-A) and Depression (HADS-D) subscales:

****0 - 7:**** Normal

****8 - 10:**** Borderline / Mild Case

****11 - 14:**** Moderate Case

****15 - 21:**** Severe Case

### ****Supplementary Table 3:Zarit Caregiver Burden Interview (ZBI)****

****Introduction:****
The Zarit Caregiver Burden Interview (ZBI) is a widely used self-report instrument that measures the subjective burden experienced by caregivers of individuals with chronic illnesses or disabilities. It assesses various aspects of burden, including health, psychological well-being, finances, social life, and the relationship between the caregiver and the care recipient^[3]^.

****Instructions for the Caregiver:****
Please read each of the following statements below and circle the response that best describes how you feel. Please indicate how often you feel this way: ****Never, Rarely, Sometimes, Quite Frequently, or Nearly Always****. There are no right or wrong answers.

| **Item No.** | **Question** | **Response Choices** |
| --- | --- | --- |
| 1 | Do you feel that your relative asks for more help than he/she needs? | 0. Never 1. Rarely 2. Sometimes 3. Quite Frequently 4. Nearly Always |
| 2 | Do you feel that because of the time you spend with your relative that you don't have enough time for yourself? | 0. Never 1. Rarely 2. Sometimes 3. Quite Frequently 4. Nearly Always |
| 3 | Do you feel stressed between caring for your relative and trying to meet other responsibilities (work/family)? | 0. Never 1. Rarely 2. Sometimes 3. Quite Frequently 4. Nearly Always |
| 4 | Do you feel embarrassed over your relative's behavior? | 0. Never 1. Rarely 2. Sometimes 3. Quite Frequently 4. Nearly Always |
| 5 | Do you feel angry when you are around your relative? | 0. Never 1. Rarely 2. Sometimes 3. Quite Frequently 4. Nearly Always |
| 6 | Do you feel that your relative currently affects your relationships with other family members or friends in a negative way? | 0. Never 1. Rarely 2. Sometimes 3. Quite Frequently 4. Nearly Always |
| 7 | Are you afraid of what the future holds for your relative? | 0. Never 1. Rarely 2. Sometimes 3. Quite Frequently 4. Nearly Always |
| 8 | Do you feel your relative is dependent upon you? | 0. Never 1. Rarely 2. Sometimes 3. Quite Frequently 4. Nearly Always |
| 9 | Do you feel strained when you are around your relative? | 0. Never 1. Rarely 2. Sometimes 3. Quite Frequently 4. Nearly Always |
| 10 | Do you feel your health has suffered because of your involvement with your relative? | 0. Never 1. Rarely 2. Sometimes 3. Quite Frequently 4. Nearly Always |
| 11 | Do you feel that you don't have as much privacy as you would like because of your relative? | 0. Never 1. Rarely 2. Sometimes 3. Quite Frequently 4. Nearly Always |
| 12 | Do you feel that your social life has suffered because you are caring for your relative? | 0. Never 1. Rarely 2. Sometimes 3. Quite Frequently 4. Nearly Always |
| 13 | Do you feel uncomfortable about having friends over because of your relative? | 0. Never 1. Rarely 2. Sometimes 3. Quite Frequently 4. Nearly Always |
| 14 | Do you feel that your relative seems to expect you to take care of him/her as if you were the only one he/she could depend on? | 0. Never 1. Rarely 2. Sometimes 3. Quite Frequently 4. Nearly Always |
| 15 | Do you feel that you don't have enough money to take care of your relative in addition to the rest of your expenses? | 0. Never 1. Rarely 2. Sometimes 3. Quite Frequently 4. Nearly Always |
| 16 | Do you feel that you will be unable to take care of your relative much longer? | 0. Never 1. Rarely 2. Sometimes 3. Quite Frequently 4. Nearly Always |
| 17 | Do you feel you have lost control of your life since your relative's illness? | 0. Never 1. Rarely 2. Sometimes 3. Quite Frequently 4. Nearly Always |
| 18 | Do you wish you could leave the care of your relative to someone else? | 0. Never 1. Rarely 2. Sometimes 3. Quite Frequently 4. Nearly Always |
| 19 | Do you feel uncertain about what to do about your relative? | 0. Never 1. Rarely 2. Sometimes 3. Quite Frequently 4. Nearly Always |
| 20 | Do you feel you should be doing more for your relative? | 0. Never 1. Rarely 2. Sometimes 3. Quite Frequently 4. Nearly Always |
| 21 | Do you feel you could do a better job in caring for your relative? | 0. Never 1. Rarely 2. Sometimes 3. Quite Frequently 4. Nearly Always |
| 22 | Overall, how burdened do you feel in caring for your relative? | 0. Never 1. Rarely 2. Sometimes 3. Quite Frequently 4. Nearly Always |

The Zarit Burden Interview (ZBI) demonstrated good internal consistency, with a total Cronbach's α of 0.870. The scale comprises two dimensions: ****Personal Strain**** and ****Role Strain****.

The ****Personal Strain**** dimension consists of items 1, 4, 5, 6, 9, 14, 16, 17, 18, 19, 20, and 21.

The ****Role Strain**** dimension consists of items 2, 3, 7, 8, 11, 12, and 13.

Item 22 (ZBI-22) measures the caregiver's perception of the ****overall burden of care****.

The total score is interpreted as follows:

A score ****≤ 40**** indicates ****No-to-Mild Burden****.

A score ****between 41 and 60**** indicates ****Moderate Burden****.

A score ****≥ 61**** indicates ****Severe Burden****.

****Supplementary Table 4:Supportive Care Needs Survey, SCNS-34****

|  | ****Not applicable**** | ****Satisfied**** | ****Low need**** | ****Moderate need**** | ****High need**** |
| --- | --- | --- | --- | --- | --- |
| 1.Pain |  |  |  |  |  |
| 2.Tiredness |  |  |  |  |  |
| 3.Feeling unwell |  |  |  |  |  |
| 4.Help with things around the house |  |  |  |  |  |
| 5.Not being able to do the things you used to do |  |  |  |  |  |
| 6.Anxiety |  |  |  |  |  |
| 7.Feeling down or depressed |  |  |  |  |  |
| 8.Feelings of sadness |  |  |  |  |  |
| 9.Fears about the cancer spreading |  |  |  |  |  |
| 10.Worry that the treatment is not working |  |  |  |  |  |
| 11.Feeling of uncertainty about the future |  |  |  |  |  |
| 12.Learning to feel in control of your situation |  |  |  |  |  |
| 13.Keeping a positive appearance |  |  |  |  |  |
| 14.Concerns about dying |  |  |  |  |  |
| 15.Worry about the worries of those close to you |  |  |  |  |  |
| 16.Changes in sexual feelings |  |  |  |  |  |
| 17.Changes in your sexual relationship |  |  |  |  |  |
| 18.Information about sexual relationships |  |  |  |  |  |
| 19.More choice about which cancer specialists you see |  |  |  |  |  |
| 20.More choice about which hospital you attend |  |  |  |  |  |
| 21.Healthcare staff acknowledging and understanding your feelings |  |  |  |  |  |
| 22.Healthcare staff attending promptly to your physical needs |  |  |  |  |  |
| 23.Healthcare staff showing sincere concern for your feelings and emotional needs |  |  |  |  |  |
| 24.Being given written information about your disease and its treatment |  |  |  |  |  |
| 25.Being given written information about managing side effects and discomfort (e.g., booklets, charts, diagrams) |  |  |  |  |  |
| 26.Being given explanations of tests |  |  |  |  |  |
| 27. Being informed about the benefits and side effects of treatments before you choose one |  |  |  |  |  |
| 28.Being informed about your test results as soon as feasible |  |  |  |  |  |
| 29.Being told your cancer is under control or in remission |  |  |  |  |  |
| 30.Guidance on what you can do to help yourself get well |  |  |  |  |  |
| 31. Access to professional counseling (e.g., psychologist, social worker, oncology nurse) for yourself or your family/friends when needed |  |  |  |  |  |
| 32. Being treated like a person, not just another case |  |  |  |  |  |
| 33.Receiving treatment in a hospital with a pleasant environment |  |  |  |  |  |
| 34.Having one hospital staff member to talk to about all aspects of your condition, treatment, and follow-up |  |  |  |  |  |

In this study, responses on the scale—"Satisfied," "Low need," "Moderate need," and "High need"—were assigned scores of 0, 1, 2, and 3, respectively. The questionnaire was administered to each patient at different follow-up time points throughout their treatment period. The mean total score and the mean scores for each of the five subscales were then calculated for each patient; these mean values were used as the final data for statistical analysis^[4]^.

Based on the final computed dataset, patients were categorized into four groups using the quartile method: "Low Need Group," "Medium-low Need Group," "Medium-high Need Group," and "High Need Group."

****Supplementary Table 5:Proportional hazards assumption tests****

| **Model** | **Variable** | **Chi-square** | **df** | **P value** |
| --- | --- | --- | --- | --- |
| Baseline Cox, continuous SCNS | SCNS total score, per 1 SD | 4.29 | 1 | 0.038 |
| Baseline Cox, continuous SCNS | HADS, per 1 SD | 5.12 | 1 | 0.024 |
| Baseline Cox, continuous SCNS | KPS, per 1 SD | 1.49 | 1 | 0.223 |
| Baseline Cox, continuous SCNS | Zarit, per 1 SD | 6.66 | 1 | 0.010 |
| Baseline Cox, continuous SCNS | education | 6.11 | 4 | 0.191 |
| Baseline Cox, continuous SCNS | economic_burden | 0.66 | 2 | 0.718 |
| Baseline Cox, continuous SCNS | afp | 1.95 | 1 | 0.162 |
| Baseline Cox, continuous SCNS | tumor_diameter | 4.57 | 1 | 0.033 |
| Baseline Cox, continuous SCNS | child_pugh | 3.05 | 1 | 0.081 |
| Baseline Cox, continuous SCNS | GLOBAL | 31.38 | 13 | 0.003 |
| Time-dependent Cox, continuous SCNS, response stratified | SCNS total score, per 1 SD | 1.24 | 1 | 0.266 |
| Time-dependent Cox, continuous SCNS, response stratified | HADS, per 1 SD | 5.50 | 1 | 0.019 |
| Time-dependent Cox, continuous SCNS, response stratified | KPS, per 1 SD | 2.67 | 1 | 0.102 |
| Time-dependent Cox, continuous SCNS, response stratified | Zarit, per 1 SD | 1.12 | 1 | 0.290 |
| Time-dependent Cox, continuous SCNS, response stratified | education | 7.37 | 4 | 0.117 |
| Time-dependent Cox, continuous SCNS, response stratified | economic_burden | 2.94 | 2 | 0.230 |
| Time-dependent Cox, continuous SCNS, response stratified | afp | 0.34 | 1 | 0.560 |
| Time-dependent Cox, continuous SCNS, response stratified | tumor_diameter | 4.26 | 1 | 0.039 |
| Time-dependent Cox, continuous SCNS, response stratified | child_pugh | 4.24 | 1 | 0.040 |
| Time-dependent Cox, continuous SCNS, response stratified | GLOBAL | 42.05 | 13 | <0.001 |
| Lagged Cox, response stratified | Previous SCNS total score, per 1 SD | 1.12 | 1 | 0.290 |
| Lagged Cox, response stratified | Previous HADS, per 1 SD | 2.20 | 1 | 0.138 |
| Lagged Cox, response stratified | Previous KPS, per 1 SD | 1.38 | 1 | 0.239 |
| Lagged Cox, response stratified | Previous Zarit, per 1 SD | 3.27 | 1 | 0.071 |
| Lagged Cox, response stratified | education | 6.21 | 4 | 0.184 |
| Lagged Cox, response stratified | economic_burden | 2.37 | 2 | 0.306 |
| Lagged Cox, response stratified | afp | 1.00 | 1 | 0.317 |
| Lagged Cox, response stratified | tumor_diameter | 3.68 | 1 | 0.055 |
| Lagged Cox, response stratified | child_pugh | 1.89 | 1 | 0.169 |
| Lagged Cox, response stratified | GLOBAL | 22.77 | 13 | 0.044 |

**Supplementary Table 6:Extended fully adjusted linear mixed-effects model for SCNS total score**

| **Variable** | **β (95% CI)** | **P value** |
| --- | --- | --- |
| Follow-up time, per 6 months | 1.08 (0.76 to 1.40) | <0.001 |
| Treatment response: PR | -1.47 (-2.08 to -0.86) | <0.001 |
| Treatment response: SD | -0.42 (-0.85 to 0.02) | 0.063 |
| Treatment response: PD | 2.52 (1.81 to 3.23) | <0.001 |
| HADS score | 1.25 (0.76 to 1.74) | <0.001 |
| KPS score | -2.51 (-3.02 to -2.01) | <0.001 |
| Zarit caregiver burden score | 0.88 (0.42 to 1.33) | <0.001 |
| Primary school | 0.01 (-5.41 to 5.42) | 0.998 |
| Junior high school | -2.89 (-8.93 to 3.15) | 0.346 |
| Senior high school | 11.47 (5.09 to 17.86) | <0.001 |
| Undergraduate college | 6.37 (-0.38 to 13.11) | 0.064 |
| Economic burden: Medium | -8.90 (-13.86 to -3.93) | <0.001 |
| Economic burden: High | -7.39 (-12.38 to -2.41) | 0.004 |
| AFP(>400ng/mL) | 5.04 (-0.63 to 10.70) | 0.081 |
| Tumor diameter(>10cm) | 5.08 (-1.89 to 12.06) | 0.152 |
| Child-Pugh(Grade B) | -6.42 (-13.69 to 0.84) | 0.083 |
| Age(>65 years) | -6.08 (-10.09 to -2.06) | 0.003 |
| Sex: Male | 1.04 (-3.45 to 5.54) | 0.647 |
| BMI: Below normal range | -0.06 (-3.97 to 3.85) | 0.974 |
| Marital status: Married | 3.19 (-2.11 to 8.49) | 0.236 |
| Religious belief: Yes | -0.22 (-4.93 to 4.48) | 0.925 |
| Place of residence: Urban | -1.41 (-5.39 to 2.57) | 0.486 |

**Supplementary Table 7:Significant findings from extended fully adjusted linear mixed-effects models for SCNS domains**

| **Outcome** | **Variable** | **β (95% CI)** | **P value** |
| --- | --- | --- | --- |
| Physical and daily living needs | Follow-up time, per 6 months | 0.10 (0.00 to 0.19) | 0.048 |
| Physical and daily living needs | Treatment response: PD | 0.61 (0.35 to 0.87) | <0.001 |
| Physical and daily living needs | HADS score | 0.36 (0.19 to 0.54) | <0.001 |
| Physical and daily living needs | KPS score | -0.58 (-0.76 to -0.40) | <0.001 |
| Physical and daily living needs | Zarit caregiver burden score | 0.24 (0.07 to 0.40) | 0.004 |
| Physical and daily living needs | Senior high school | 2.54 (0.60 to 4.47) | 0.011 |
| Physical and daily living needs | Place of residence: Urban | -1.53 (-2.73 to -0.33) | 0.013 |
| Psychological needs | Follow-up time, per 6 months | 0.35 (0.21 to 0.48) | <0.001 |
| Psychological needs | Treatment response: PR | -0.38 (-0.64 to -0.11) | 0.006 |
| Psychological needs | Treatment response: PD | 0.74 (0.44 to 1.05) | <0.001 |
| Psychological needs | HADS score | 0.72 (0.50 to 0.93) | <0.001 |
| Psychological needs | KPS score | -0.74 (-0.96 to -0.52) | <0.001 |
| Psychological needs | Senior high school | 4.65 (1.17 to 8.14) | 0.009 |
| Psychological needs | Undergraduate college | 6.47 (2.79 to 10.16) | <0.001 |
| Psychological needs | AFP(>400ng/mL) | 3.68 (0.58 to 6.77) | 0.020 |
| Psychological needs | Age(>65 years) | -3.63 (-5.83 to -1.44) | 0.001 |
| Sexual needs | Treatment response: PD | 0.45 (0.23 to 0.68) | <0.001 |
| Sexual needs | Zarit caregiver burden score | 0.21 (0.08 to 0.34) | 0.002 |
| Patient care and support needs | Treatment response: PR | -0.40 (-0.62 to -0.18) | <0.001 |
| Patient care and support needs | Treatment response: SD | -0.17 (-0.33 to -0.02) | 0.028 |
| Patient care and support needs | Treatment response: PD | 0.42 (0.17 to 0.67) | 0.001 |
| Patient care and support needs | HADS score | 0.37 (0.20 to 0.54) | <0.001 |
| Patient care and support needs | KPS score | -0.29 (-0.46 to -0.11) | 0.002 |
| Patient care and support needs | Zarit caregiver burden score | 0.28 (0.12 to 0.44) | <0.001 |
| Patient care and support needs | Senior high school | 2.32 (0.32 to 4.32) | 0.023 |
| Patient care and support needs | Undergraduate college | 3.43 (1.32 to 5.55) | 0.002 |
| Patient care and support needs | Economic burden: High | -2.44 (-3.99 to -0.90) | 0.002 |
| Patient care and support needs | Religious belief: Yes | 1.48 (0.01 to 2.95) | 0.049 |
| Health system and information needs | Follow-up time, per 6 months | 0.26 (0.14 to 0.37) | <0.001 |
| Health system and information needs | Treatment response: PR | -0.32 (-0.57 to -0.08) | 0.010 |
| Health system and information needs | Treatment response: PD | 0.59 (0.30 to 0.87) | <0.001 |
| Health system and information needs | KPS score | -0.78 (-0.99 to -0.58) | <0.001 |
| Health system and information needs | Economic burden: Medium | -6.06 (-9.79 to -2.33) | 0.002 |
| Health system and information needs | Economic burden: High | -4.47 (-8.21 to -0.73) | 0.019 |
| Health system and information needs | Sex: Male | 4.55 (1.18 to 7.92) | 0.008 |
| Health system and information needs | Marital status: Married | 4.84 (0.87 to 8.81) | 0.017 |

**Supplementary Table 8:Model information for extended fully adjusted sensitivity analyses**

| **Outcome** | **Model** | **Singular fit** | **AIC** | **BIC** | **logLik** |
| --- | --- | --- | --- | --- | --- |
| SCNS total score | Extended LMM random slope | FALSE | 3631.1 | 3752.7 | -1788.6 |
| Physical and daily living needs | Extended LMM random slope | FALSE | 2179.7 | 2301.3 | -1062.8 |
| Psychological needs | Extended LMM random slope | FALSE | 2573.5 | 2695.0 | -1259.7 |
| Sexual needs | Extended LMM random slope | FALSE | 1858.3 | 1979.8 | -902.1 |
| Patient care and support needs | Extended LMM random slope | FALSE | 2188.7 | 2310.3 | -1067.3 |
| Health system and information needs | Extended LMM random slope | FALSE | 2581.8 | 2703.4 | -1263.9 |

The extended sensitivity models included all covariates in the primary model and additionally adjusted for age, sex, BMI, marital status, religious belief, and place of residence. SCNS=Supportive Care Needs Survey; HADS=Hospital Anxiety and Depression Scale; KPS=Karnofsky Performance Status; AFP=alpha-fetoprotein.

**References**

[1]Karnofsky, D. A., & Burchenal, J. H. (1949). The clinical evaluation of chemotherapeutic agents in cancer. In C. M. MacLeod (Ed.), Evaluation of chemotherapeutic agents (pp. 191–205). Columbia University Press.

[2]Zigmond, A. S., & Snaith, R. P. (1983). The hospital anxiety and depression scale. Acta Psychiatrica Scandinavica, 67(6), 361–370.

[3]Zarit, S. H., Reever, K. E., & Bach-Peterson, J. (1980). Relatives of the impaired elderly: correlates of feelings of burden. The Gerontologist, 20(6), 649–655.

[4]Bonevish, H., et al. (1999). The Supportive Care Needs Survey: A guide to administration, scoring and analysis. Newcastle, Australia: Centre for Health Research & Psycho-oncology.
